# Unlocking Value: A Cost-Effectiveness Analysis of Hip PJI Diagnostic Pathways Employing the European Bone and Joint Infection Society/Musculoskeletal Infection Society Definition

**DOI:** 10.1016/j.artd.2026.102004

**Published:** 2026-04-04

**Authors:** Claudio Diaz-Ledezma, Ahmad M. Zedan, Angel Xiao, Thomas Barber

**Affiliations:** a Department of Orthopaedic Surgery, University of California San Francisco, San Francisco, CA, USA; b University of California San Francisco, School of Medicine, San Francisco, CA, USA

**Keywords:** Periprosthetic joint infection, Total hip arthroplasty, Cost-effectiveness analysis, EBJIS/MSIS criteria, Diagnosis

## Abstract

**Background:**

The European Bone and Joint Infection Society (EBJIS)/Musculoskeletal Infection Society (MSIS) criteria provide a framework for diagnosing hip periprosthetic joint infection, yet daily clinical application lacks a standardized algorithm. Since these criteria rely heavily on synovial fluid markers, surgeons encounter 2 primary dilemmas: the reliability of aspiration techniques (ultrasound vs fluoroscopy) and the economic trade-off between higher-cost proprietary assays and standard institutional laboratories. This cost-effectiveness analysis was conducted to identify the diagnostic pathway that maximizes clinical benefit while minimizing expenditure within the EBJIS/MSIS framework.

**Methods:**

A decision tree model simulated the diagnostic workup of a 67-year-old patient with suspected hip periprosthetic joint infection. Ten strategies were evaluated, comparing aspiration modality and laboratory method (institutional, point-of-care, and third-party analysis). The model incorporated 1- and 2-stage revision pathways and included dry tap scenarios. Accuracy inputs were derived from the literature, and costs reflected 2025 Medicare schedules. Sensitivity analyses tested robustness across diagnostic outcomes. Effectiveness was measured in quality-adjusted life years based on downstream surgical outcomes.

**Results:**

Ultrasound-guided aspiration with third-party synovial analysis was most cost-effective in both revision scenarios. Despite higher upfront costs of specialized testing, its superior sensitivity and specificity maximized quality-adjusted life years by reducing morbidity from misdiagnosis. Institutional testing was preferred only if sensitivity exceeded 90% and specificity 94%. In “dry tap” cases, intraoperative lateral flow alpha-defensin testing was favored over preoperative nuclear imaging. Surgical outcome utility most strongly influenced the results.

**Conclusions:**

Within the EBJIS/MSIS framework, prioritizing high-accuracy diagnostic tools maximizes value by reducing misaligned revision strategies. Diagnostic precision outweighed individual test cost in determining cost-effectiveness.

**Level of Evidence:**

Economic and decision analysis, Level II.

## Introduction

Periprosthetic joint infection (PJI) remains a devastating complication of total hip arthroplasty. Although several diagnostic frameworks have been proposed—including those from the 2018 International Consensus Meeting (ICM) [1] and the 2013 Infectious Diseases Society of America [2] —the more recent definition developed jointly by the European Bone and Joint Infection Society (EBJIS) and the American Musculoskeletal Infection Society (MSIS) [3] has gained widespread recognition because of its ability to reduce indeterminate diagnoses and improve clinical decision-making [4,5]. Integrating this framework within a value-based healthcare environment remains an ongoing challenge [6].

Synovial fluid analysis obtained through hip aspiration is a central component of the EBJIS/MSIS criteria. However, 2 important challenges arise in routine practice. First, the optimal technique for hip aspiration remains unclear. There is no consensus on whether ultrasound- or fluoroscopy-guided aspiration provides superior diagnostic yield or cost-effectiveness, particularly given the risk of a “dry tap.” Second, the EBJIS/MSIS framework assigns equal diagnostic weight to multiple synovial assays, including proprietary commercial tests (eg, Synovasure, Zimmer Biomet, Warsaw, IN) that carry substantially higher upfront costs than institutional laboratory analyses. Whether these additional costs translate into clinically meaningful improvements in diagnostic accuracy and long-term outcomes remains uncertain

Because serum marker screening is not included in the EBJIS/MSIS diagnostic algorithm, joint aspiration with synovial fluid analysis is required in all patients undergoing revision for suspected PJI. These features raise important questions regarding resource utilization and cost-effectiveness within a value-based healthcare environment. Clarifying the most efficient and economically responsible diagnostic strategy is therefore essential.

The present study evaluated the cost-effectiveness of 10 distinct preoperative diagnostic strategies within the EBJIS/MSIS framework. Specifically, we sought to determine which strategy is most cost-effective for confirming hip PJI before 1-stage vs 2-stage revision, how pretest probability influences comparative performance, and which model variables most strongly affect cost-effectiveness outcomes.

## Material and methods

### Model overview

**Figure 1:**
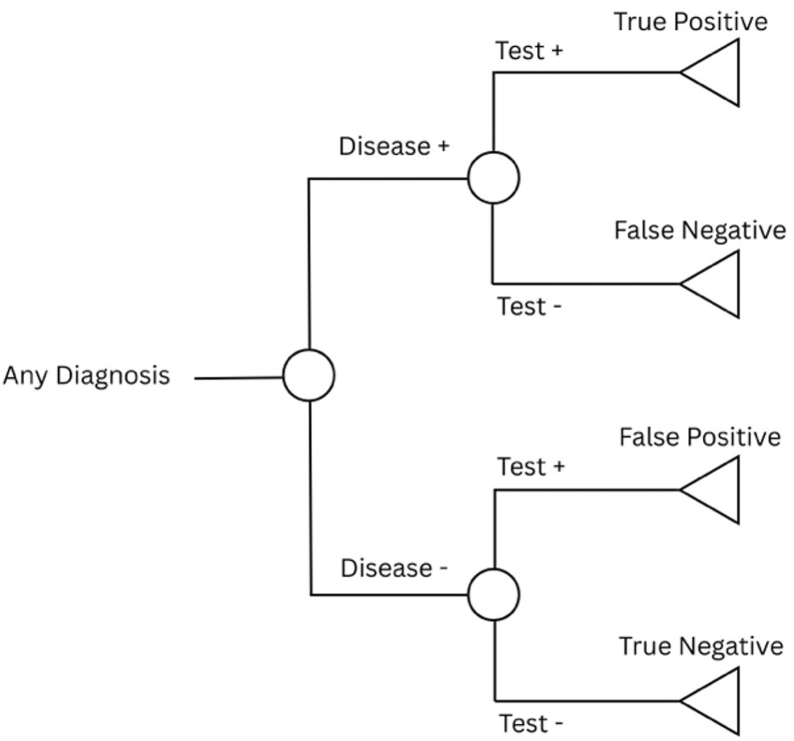
Diagnostic framework overview. Simplified decision tree structure illustrating diagnostic outcomes based on true disease status and test result. Patients with and without disease may test positive or negative, resulting in one of four diagnostic categories: true positive, false negative, false positive, or true negative. This model forms the foundation of the cost-effectiveness analysis performed in the study.

Given the lack of consensus on methodologies for economic evaluations of diagnostic tests [7], we employed decision analysis using decision tree models [8]. Decision tree models are an established and recommended method for evaluating the cost-effectiveness of diagnostic strategies [7] and have precedent in the hip PJI literature [9]. A disease-based modeling approach was used [10] (Fig. 1), guided by the recommendations of the Panel on Cost-Effectiveness in Health and Medicine [11,12].

Two decision tree models were created to evaluate the diagnostic workup of hip PJI: Track 1: The first decision tree was for a 67-year-old patient with a history of a cementless total hip arthroplasty performed 24 months ago. This individual is presenting to clinic with a painful joint for the last 4 months and radiographic loosening of the femoral implant. No sinus tracts were encountered at physical examination, and the surgeon who is seeing the patient would be willing to perform a 1-stage revision in case PJI is confirmed. Track 2: The second decision tree was for the same patient visiting a surgeon who only performs 2-stage revisions. Both models were programmed in TreeAge Pro (2024 version R2.1, TreeAge Software Inc.).

The primary outcome was the incremental cost-effectiveness ratio (ICER), defined as the difference in cost between strategies divided by the difference in health benefit. Health benefit was measured in quality-adjusted life years (QALYs), calculated as the product of health state utility and duration. Utility values range from 0 (death) to 1 (perfect health) and reflect patient preference for a given health state. This study did not involve human subjects or patient-level data; therefore, institutional review board approval was not required.

### Diagnostic strategies

Diagnostic strategies were selected to reflect all available approaches for fulfilling confirmatory EBJIS/MSIS criteria either preoperatively or intraoperatively when preoperative confirmation was not achieved. All patients entering the model were assumed to have a clinically reasonable suspicion of chronic PJI (“*infection likely”* status), defined as radiographic implant loosening within 5 years of the index procedure, before undergoing revision surgery.

Disease status (PJI present or absent) was assigned at model entry and remained fixed throughout the analysis. The model horizon, or specific time frame covered by the decision tree, was the diagnostic period that culminated with either a confirmed or unconfirmed PJI case, leading to different treatment options. Intraoperative frozen section analysis was excluded due to variability in availability and implementation across institutions, although its diagnostic role is acknowledged.

**Figure 2:**
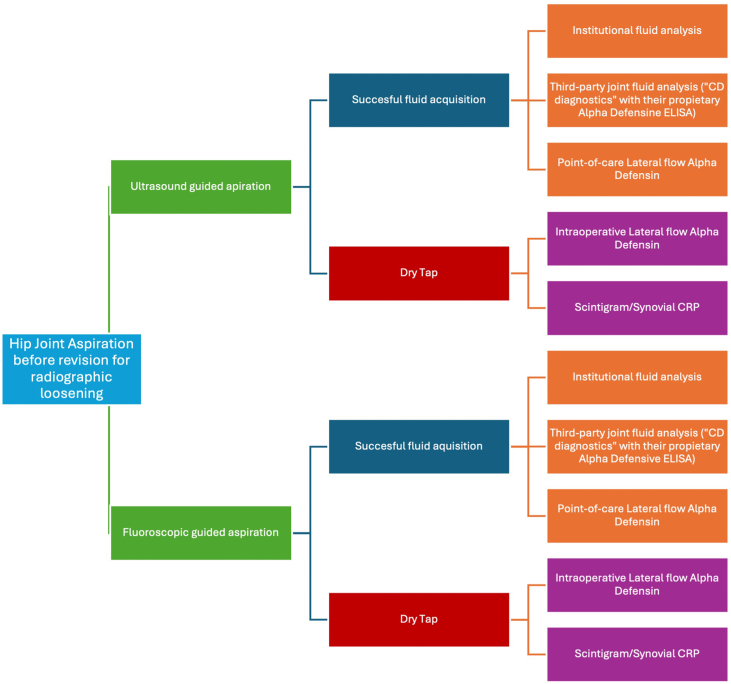
Diagnostic strategy mapping across aspiration modality and fluid analysis techniques. This model shows the 10 diagnostic strategies evaluated in the cost-effectiveness analysis of hip periprosthetic joint infection (PJI). Strategies are grouped by aspiration technique (ultrasound vs fluoroscopy) and type of synovial fluid analysis (institutional, point-of-care lateral flow alpha-defensin, or third-party). Pathways for managing dry tap events are also depicted, including fallback to preoperative CRP and WBC-labeled scintigraphy or intraoperative alpha-defensin testing.

Ten diagnostic strategies were evaluated using a 2-level decision tree (Fig. 2). These strategies represented all combinations of 2 aspiration techniques (ultrasound-guided and fluoroscopy-guided) and 3 synovial fluid analysis methods (institutional laboratory analysis, point-of-care Synovasure lateral flow test [LFAD, Zimmer Biomet, Warsaw, IN], and third-party analysis via Synovasure). Only tests included within the EBJIS/MSIS criteria were incorporated. In cases of dry tap, 2 management approaches were compared: (1) a preoperative step-back strategy consisting of white blood cell (WBC)-labeled scintigraphy plus serum C-reactive protein (CRP) (maintaining “infection likely” status), and (2) intraoperative confirmation using point-of-care LFAD.

### Clinical data

Clinical parameter estimates are summarized in Table 1. A structured literature review was conducted to identify data for all model inputs, including PJI prevalence among patients undergoing diagnostic arthrocentesis, aspiration success rates by technique, and sensitivity and specificity of each diagnostic test. For each parameter, the study with the most robust methodology and greatest applicability to the model population was selected.

**Table 1:** References and values for the aspiration strategies compared in this study.

Test/Parameter	Sensitivity (95% CI)	Specificity (95% CI)	Value/Range	Reference
Diagnostic tests				
PMN% (synovial fluid)	0.86 (0.76 - 0.92)	0.89 (0.75 - 0.96)	-	Goud JOA 2022 [13]
WBC (synovial fluid)	0.88 (0.72 - 0.95)	0.91 (0.82 - 0.95)	-	Goud JOA 2022 [13]
Preoperative culture	0.63 (0.56 - 0.70)	0.96 (0.93 - 0.98)	-	Watanabe JOA 2024 [14]
WBC scintigraphy	0.88 (0.81 - 0.94)	0.92 (0.88 - 0.96)	-	Verberne JBJS 2016 [15]
CRP serum	0.77 (0.72 - 0.82)	0.80 (0.76 - 0.83)	-	Goud JOA 2022 [13]
Alpha defensin lateral flow	0.86 (0.82 - 0.9)	0.97 (0.94 - 0.98)	-	Kuiper CORR 2020 [16]
Alpha defensin immunoassay	0.9 (0.84 - 0.95)	0.96 (0.94 - 0.97)	-	Kuiper CORR 2020 [16]
Model parameters				
Prevalence of PJI	-	-	0.19 (0.14 - 0.63)	Deirmengian JBJS 2021 [17], Goud JOA 2022 [13]
Fluoroscopy-guided aspiration success rate	-	-	0.59 (0.53 - 0.65)	Christensen, JOA 2022 [18], Treu J Bone Jt Infect 2023 [19], Kanthawang, Skelet Radiol 2021 [20]
Ultrasound-guided aspiration success rate	-	-	0.76 (0.65 - 0.85)	Christensen, JOA 2022 [18], Treu J Bone Jt Infect 2023 [19], Duck, J Bone Jt Infect 2021 [21]
Possibility of PJI in a hip dry tap			0.16	Treu JOA 2024 [22]

Outcome description	QALY value	Reference	Notes
Success after one-stage revision for PJI	0.75	Blom [23]	True positive (one-stage)
Requiring septic re-revision after failing one-stage (failure after septic revision)	0.46	Bozic [24]	False positive (one-stage)
Success after two-stage revision for PJI	0.69	Blom [23]	True positive (two-stage)
Requiring septic re-revision after failing two-stage (failure after septic revision)	0.46	Bozic [24]	False positive (two-stage)
Requiring re-revision due to infection after previous aseptic revision	0.58	Konopka [25]	False negative
Not requiring re-revision after aseptic revision	0.8	Konopka [25]	True negative

This table summarizes the sensitivity, specificity, and estimated ranges for diagnostic tests and model parameters used in the decision tree analysis of periprosthetic joint infection (PJI) in total hip arthroplasty (THA).

PMN%, polymorphonuclear cell percentage; CIs, confidence intervals.

PJI prevalence was set at 19% based on Deirmengian et al. [17], as they demonstrated diagnostic performance of the alpha defensin test and directly supported Food and Drug Administration authorization of the lateral-flow test as an aid in diagnosing PJI, underscoring the rigor and clinical relevance of their methodology. A confidence interval of 14-63% was derived from the study by Goud et al [13], which provided the most robust estimate of uncertainty around this prevalence. Although the upper limit of this interval suggests a much higher prevalence, we considered the 19% point estimate most appropriate for modeling purposes as it reflects the best available evidence from a large, methodologically rigorous study and avoids underestimation or overestimation reported in smaller investigations. With regard to the diagnostic tests evaluated, we chose systematic reviews and high-quality series that described the sensitivity and specificity of each test as well as their confidence intervals for sensitivity analysis. For strategies using multiple tests, we assumed synergy between tests. Thus, the best sensitivity and the best specificity were used to calculate the strategy accuracy.

### Cost data

Cost inputs are presented in Table 2. All costs were evaluated from the payer perspective and expressed in 2025 US dollars. National average costs for diagnostic laboratory tests were sourced from the Medicare Clinical Laboratory Fee Schedule. The costs associated with diagnostic interventions identified by a Current Procedural Terminology code were sourced from the Physician Fee Schedule lookup tool on the U.S. Centers for Medicare & Medicaid Services website. The cost for The Synovasure Comprehensive PJI Panel (Proprietary Test) including their Synovasure alpha-defensin immunoassay (ADIA, Zimmer Biomet, Warsaw, IN) was obtained from the company's website, and the cost for their proprietary point-of-care LFAD test was obtained from our institution. The total cost for each diagnostic strategy was calculated by summing the individual costs of all relevant diagnostic laboratory tests and/or interventions.

**Table 2:** Cost estimates for diagnostic strategies in the evaluation of suspected Hip PJI.

Procedure	CPT code(s)	Cost (USD)	Reference/Source
Ultrasound-guided aspiration	20611	$339.00	https://www.medicare.gov/procedure-price-lookup/
Fluoroscopy-guided aspiration	77002 + 20610	$440.89	https://www.medicare.gov/procedure-price-lookup/
WBC scintigraphy	78102	$415.00	https://www.medicare.gov/procedure-price-lookup/
The Synovasure Comprehensive PJI Panel (Proprietary Test)	N/A	$269.00	https://cdlaboratories.com/home/patient/infection/
Lateral flow alpha-defensin (LFAD) test	N/A	$405.00	(Cost at our institution)
Two cultures (aerobic and anaerobic)	N/A	$33.40	https://www.cms.gov/medicare/payment/fee-schedules/clinical-laboratory-fee-schedule-clfs/files/25clabq1
Body fluid cell count with identification	N/A	$5.60	https://www.cms.gov/medicare/payment/fee-schedules/clinical-laboratory-fee-schedule-clfs/files/25clabq1
Serum C-reactive protein (CRP)	N/A	$5.18	https://www.cms.gov/medicare/payment/fee-schedules/clinical-laboratory-fee-schedule-clfs/files/25clabq1

Aspiration technique	Diagnostic pathway	Total cost (USD)
Fluoroscopy-guided	Institutional WBC and differential	$479.89
Fluoroscopy-guided	CD diagnostics	$709.89
Fluoroscopy-guided	LFAD + institutional cultures	$879.29
Fluoroscopy-guided	Step-back: scintigraphy and CRP	$861.00
Fluoroscopy-guided	Intraoperative LFAD confirmation	$845.00
Ultrasound-guided	Institutional WBC and differential	$378.00
Ultrasound-guided	CD diagnostics	$608.00
Ultrasound-guided	LFAD + institutional cultures	$777.40
Ultrasound-guided	Step-back: scintigraphy and CRP	$759.18
Ultrasound-guided	Intraoperative LFAD confirmation	$744.00

This table details procedural and laboratory costs used in the decision models, stratified by aspiration technique and synovial fluid analysis approach. All costs are presented in 2025 United States dollars (USD) from the Medicare payer perspective.

CPT, Current Procedural Terminology; CD diagnostics (commercial third-party laboratory performing the Synovasure Comprehensive PJI Panel).

### Cost-effectiveness analysis

The sensitivity and specificity of each confirmatory diagnostic strategy were used to calculate the proportions of true positives, false positives, true negatives, and false negatives. The health status QALYs values we chose to associate with each outcome are as follows:

• True positive: Successful septic revision (QALY 0.75 for 1-stage; 0.69 for 2-stage) [23].
• False positive: Unnecessary septic revision resulting in failure (QALY 0.46) [24].
• True negative: Successful aseptic revision (QALY 0.80) [25].
• False negative: Initial aseptic revision followed by septic rerevision (QALY 0.58) [25].

A willingness-to-pay threshold of $50,000 per QALY was used, consistent with widely accepted U.S. cost-effectiveness standards [26].

A roll-back analysis was performed to determine the optimal strategy. This analysis begins at the terminal nodes or end points of the decision tree and works backward. For each decision node, the expected value is calculated by summing the products of the probability of each outcome and their respective payoffs; the decision with the highest expected value is chosen as the optimal choice. The process repeats through the tree until the initial decision node is reached

One-way sensitivity analysis was performed to identify variables with the strongest influence on cost-effectiveness or ICER. This technique calculates the ICER across a range of input values for a single variable. Given the large number of variables in our model, we plotted one-way sensitivities of every variable using a tornado diagram. This plot displays all variables across the same ICER scale to determine which variables have the most impact. Threshold analysis was also performed to identify the value of a given variable that would cause the preferred strategy to shift from one diagnostic strategy to another. Lastly, 2-way sensitivity analysis, accounting for the variability in sensitivity and specificity observed in institutional synovial fluid analysis compared to third party testing, was performed. This technique explores how the optimal strategy changes across ranges of 2 input variables.

## Results

### Most cost-effective preoperative diagnostic strategy

**Supplementary Figure 2:**
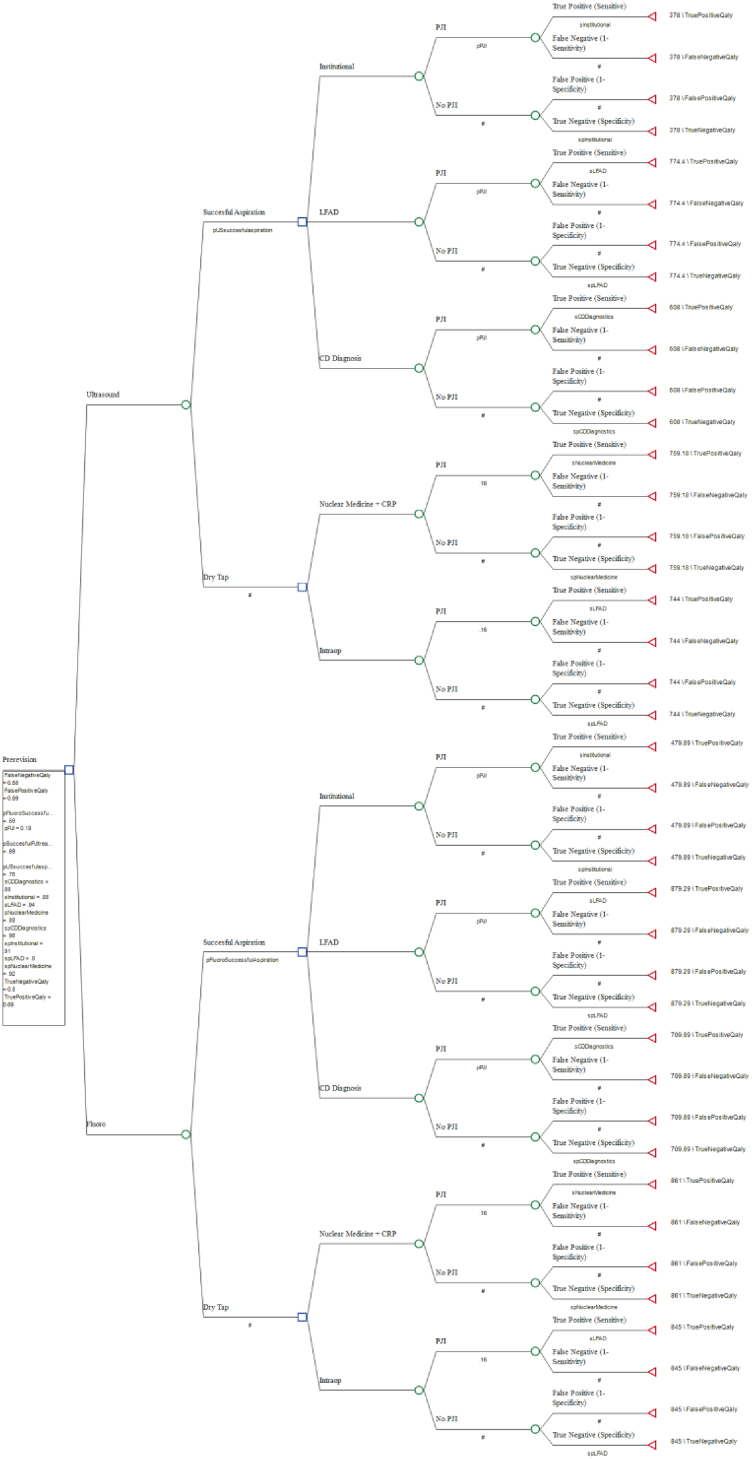
Full Decision Tree. Visualization of the ten diagnostic pathways evaluated, showing combinations of aspiration modality (ultrasound vs fluoroscopy) and synovial fluid analysis type (institutional, point-of-care lateral flow alpha-defensin, or third-party) as well as associated resultant QALY values at the terminal nodes.

In both the 1-stage and 2-stage revision track models, roll-back analysis indicated that using ultrasound-guided aspiration followed by joint fluid analysis performed by Synovasure (third-party analysis) had the highest likelihood of being cost-effective. In cases of dry tap, intraoperative LFAD testing was more cost-effective than preoperative bone scintigraphy and CRP testing. These findings were consistent across both revision strategies. The full decision tree is included in Supplementary Figure 2.

### Performance thresholds for institutional synovial fluid analysis

**Figure 3:**
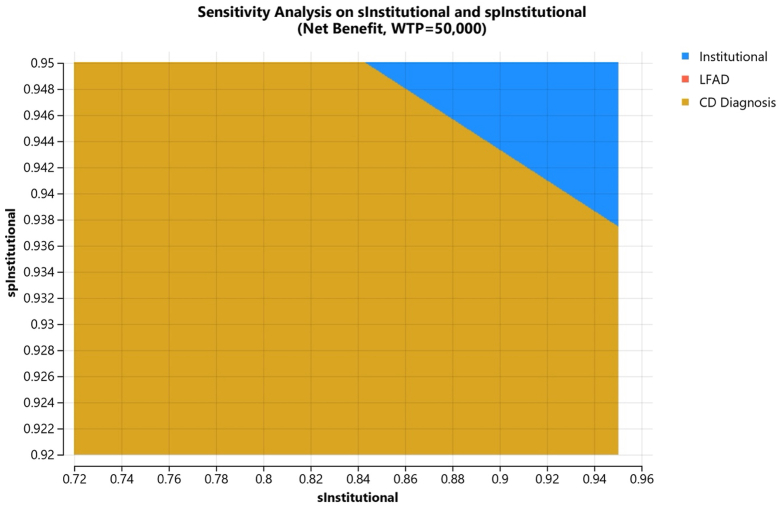
Two-way sensitivity analysis considering variability in sensitivity and specificity observed in institutional synovial fluid analysis. Graphical analysis showing how changes in sensitivity and specificity affect the cost-effectiveness of institutional synovial fluid analysis compared to third-party testing. The threshold values at which institutional analysis becomes the preferred strategy are indicated. spInstitutional, specificity of institutional diagnostic testing; sInstitutional, sensitivity of institutional diagnostic testing; WTP, willingness-to-pay.

Two-way sensitivity analyses demonstrated that for institutional synovial fluid analysis to become the most cost-effective option, it would need to achieve a sensitivity greater than 0.90 and specificity greater than 0.94. These thresholds were consistent in both the 1-stage and 2-stage revision models (Fig. 3).

### Influence of pretest probability on aspiration modality

One-way sensitivity analysis demonstrated that the pretest probability of PJI had a substantial impact on aspiration modality choice. For 1-stage revisions, fluoroscopy-guided aspiration became cost-effective only when the pretest probability exceeded 40%. For two-stage revisions, this threshold was slightly lower, at 29%. For synovial fluid analysis, a third-party conducted analysis remained the dominant strategy along all the different PJI pretest probabilities.

### Most influential model variables

**Figure 4:**
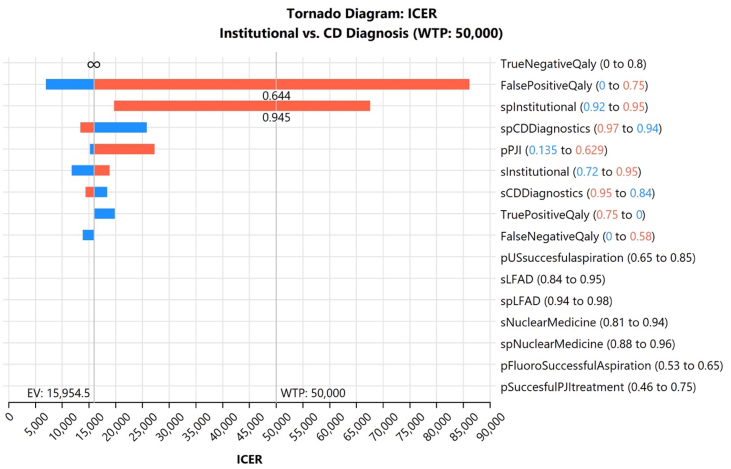
Tornado diagram of the incremental cost-effectiveness ratio (ICER) comparing institutional vs third-party fluid synovial fluid analysis at a willingness-to-pay (WTP) threshold of $50,000. Bars represent the impact of parameter uncertainty on ICER values. EV, expected value; pPJI, probability of periprosthetic joint infection; spInstitutional, specificity of institutional diagnostic testing; sInstitutional, sensitivity of institutional diagnostic testing; spCDDiagnostics, specificity of consensus definition diagnostics; sCDDiagnostics, sensitivity of consensus definition diagnostics; TrueNegativeQaly, utility (QALY) associated with a true negative diagnosis; TruePositiveQaly, utility (QALY) associated with a true positive diagnosis; FalsePositiveQaly, utility (QALY) associated with a false positive diagnosis; FalseNegativeQaly, utility (QALY) associated with a false negative diagnosis; pUSsuccessfulaspiration, probability of successful aspiration with ultrasound guidance; sLFAD, sensitivity of lateral flow alpha-defensin test; spLFAD, specificity of lateral flow alpha-defensin test; sNuclearMedicine, sensitivity of nuclear medicine imaging; spNuclearMedicine, specificity of nuclear medicine imaging; pFluoroSuccessfulAspiration, probability of successful aspiration with fluoroscopy guidance; pSuccessfulPJITreatment, probability of successful treatment of periprosthetic joint infection.

Tornado diagram analysis identified the QALYs associated with true-positive and true-negative PJI diagnoses as the most influential variables affecting model outcomes. This finding held true for both revision pathways, underscoring the importance of accurate diagnosis in driving long-term clinical benefit and economic value (Fig. 4).

## Discussion

This study employed decision tree models to evaluate the cost-effectiveness of various diagnostic strategies for hip PJI within the framework of the EBJIS/MSIS criteria, distinguishing between scenarios for 1-stage and 2-stage revision surgeries. For all patients, a third-party fluid analysis using ADIA after ultrasound-guided aspiration was the dominant diagnostic strategy. In the case of a dry tap, intraoperative LFAD proved more cost-effective than preoperative bone scintigraphy and CRP measurement.

While most studies in the field of PJI diagnosis have examined the diagnostic accuracy of individual tests, to our knowledge, this study represents the first formal evaluation of the cost-effectiveness of various diagnostic strategies for PJI, thereby addressing a critical gap in the existing literature. Our research builds on previous studies which demonstrated the value of serum screening to fulfill the MSIS 2011 PJI diagnostic criteria [27] through multicriteria decision analysis using costs, benefits, risk, and opportunities [28]. Our present findings further refine diagnostic strategies based on updated definitions and updated diagnostic techniques. Notably, the utilization of ultrasound-guided hip aspiration significantly outperformed fluoroscopically guided aspirations which, within our institutional experience, is associated with a 30% rate of dry taps [20]. Moreover, ultrasound-guided aspiration is both cheaper than fluoroscopy-guided aspiration ($339 vs $440) and has been shown in prior studies to have improved sensitivity and specificity as well as improved overall yield, making it the dominant strategy [19,21]. However, all current studies investigating ultrasound-guided aspiration come from high-volume academic arthroplasty centers. We recognize that the effectiveness of ultrasound-guided aspiration can be incredibly user-dependent and requires a higher level of training. Future studies should investigate the reproducibility of ultrasound-guided aspiration as, depending on the practice setting, this strategy may not be feasible.

Furthermore, the advantages observed in our model for synovial fluid analysis conducted by a third-party company warrant further rigorous investigation. Synovasure testing at our institution is significantly more expensive and time-consuming. This is a consistent experience across other studies who note the high sensitivity and specificity of the ADIA test, but take pause at the high cost, particularly given the comparably high sensitivity and specificity of cheaper institutional synovial tests such as WBC or polymorphonuclear cell percentage [29,30]. More importantly, there is recent evidence suggesting that the agreement of diagnostic criteria based on synovial WBC analysis can significantly vary between different laboratories [31]. We posit that the associated analytical costs are proportionally minor in the context of the potential benefits under the EBJIS/MSIS criteria. Moreover, given that the willingness-to-pay threshold is considerably higher than the observed cost differential between any diagnostic testing, any cost differences appear to be mitigated by the benefits accrued through the reduction of false-positive and false-negative results, which can lead to surgical interventions that may compromise the quality of life of our patients. Our model, however, is unable to account for the time taken to obtain Synovasure panel compared to institutional labs and thus, we are unable to account of QALY differentials between extended time in the pretreatment disease state. Our results, however, are meaningful as they indicate that other expensive tests such as three-phase bone scans are unnecessary, and their costs are not offset by improves in overall QALY.

One of the most difficult aspects of cost-effectiveness analysis (CEA) is determining the costs of providing care [32]. We chose to adopt the perspective of the payer, specifically Medicare, to input the costs associated with the various diagnostic tactics, partially employing methodology used in recent in CEA studies in the field of PJI [33]. While we acknowledge that other alternatives exist for describing costs [34], we believe that the utilization of publicly available cost data related to diagnostic tests and procedural codes represents an acceptable methodology for describing direct costs, and this constitutes a reasonable approach for our purposes.

This study's strengths include its methodology, utilizing decision tree models and comprehensive sensitivity analyses to assess the robustness of findings. The incorporation of the EBJIS/MSIS criteria and the differentiation between 1-stage and 2-stage revision scenarios enhance the clinical relevance of our results. However, several limitations warrant consideration. First, the model relies on literature-derived parameters, which may not fully reflect real-world variability. Second, cost data were based on national averages, potentially limiting the applicability to specific institutional settings. Third, the assumption of synergy between tests in combined strategies might not always hold true. Lastly, it is possible that with other diagnostic criteria such as the ICM that utilizes a more stepwise approach, a dependence on serum markers and institutional synovial analysis may prove to be more cost-effective. Future studies should compare existing diagnostic criteria, such as the ICM criteria, and potentially new diagnostic criteria, to consider cost-effectiveness in evaluating utility within the greater healthcare system. Future research should explore the impact of local cost variations and validate our findings in different clinical settings.

## Conclusions

This CEA demonstrates that ultrasound-guided aspiration combined with third-party synovial fluid analysis (Synovasure) is the most cost-effective preoperative diagnostic strategy for suspected hip PJI within the EBJIS/MSIS framework. When aspiration results in a dry tap, intraoperative LFAD testing represents the preferred alternative. By integrating diagnostic performance with economic evaluation, this study provides an evidence-based framework to support value-conscious decision-making in the workup of hip PJI to optimize resource allocation. Future investigations should validate these findings in varied clinical environments and examine how patient-level factors may further refine diagnostic pathways.

## CRediT authorship contribution statement

**Claudio Diaz-Ledezma:** Writing – original draft, Visualization, Validation, Supervision, Software, Resources, Project administration, Methodology, Investigation, Formal analysis, Data curation, Conceptualization. **Ahmad M. Zedan:** Writing – review & editing, Writing – original draft, Visualization, Validation, Project administration, Methodology. **Angel Xiao:** Writing – review & editing, Visualization, Project administration, Formal analysis, Data curation, Conceptualization. **Thomas Barber:** Writing – review & editing, Visualization, Validation, Supervision, Methodology, Conceptualization.

## Conflicts of interest

C. Diaz-Ledezma is a member of the medical/orthopaedic publications editorial/governing board at *Journal of Arthroplasty*; all other authors declare no potential conflicts of interest.

For full disclosure statements refer to https://doi.org/10.1016/j.artd.2026.102004.

## Figures and Tables

**Supplementary Figure 1:**
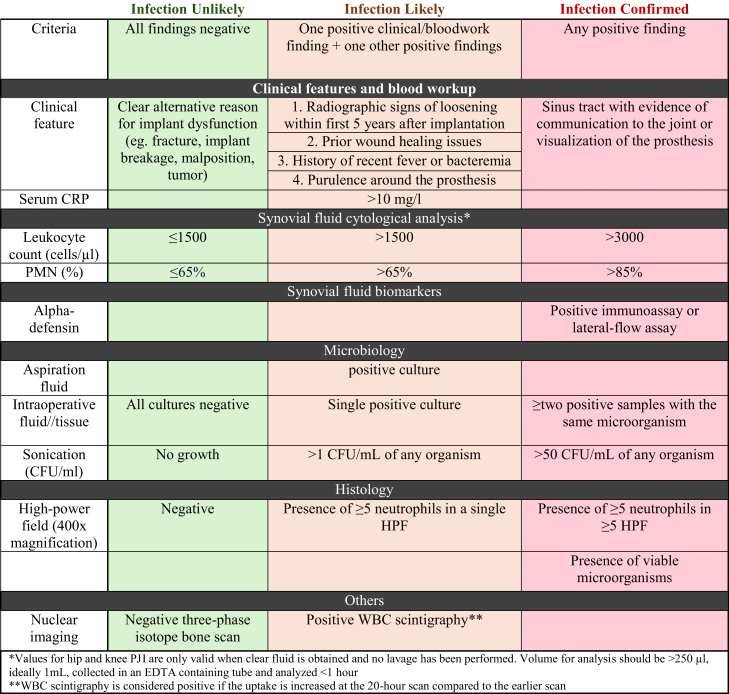
Periprosthetic joint infection diagnostic criteria as developed by the European Bone and Joint Infection Society (EBJIS), and the Musculoskeletal Infection Society (MSIS).

## Appendix A Supplementary data

Conflict of Interest Statement for Zedan

Conflict of Interest Statement for Diaz-Ledezma

Conflict of Interest Statement for Xiao

Conflict of Interest Statement for Barber
