# Supplementary material for: Unlocking Value: A Cost-Effectiveness Analysis of Hip PJI Diagnostic Pathways Employing the European Bone and Joint Infection Society/Musculoskeletal Infection Society Definition
Source: Arthroplast Today. 2026 Apr 4;39:102004. doi: 10.1016/j.artd.2026.102004 (PMC13089072; doi:10.1016/j.artd.2026.102004)
Supplement: Conflict of Interest Statement for Xiao [file mmc3.pdf]

CONFLICT OF INTEREST STATEMENT

The Journal of Arthroplasty

(Adopted from the American Academy of Orthopaedic Surgeons disclosure statement)

The following form **must be filled out completely and submitted by each author (example, 6 authors, 6 forms). If no discloser is required, please write/type "none" at the end of each sentence**.

Manuscript Title **Unlocking Value: A Cost-Effectiveness Analysis of Hip PJI Diagnostic Pathways Employing the European Bone and Joint Infection Society / Musculoskeletal Infection Society Criteria**

1. Royalties from a company or supplier (The following conflicts were disclosed)
   None
2. Speakers bureau/paid presentations for a company or supplier (The following conflicts were disclosed)
   None

3A. Paid employee for a company or supplier (The following conflicts were disclosed)
   None

3B. Paid consultant for a company or supplier (The following conflicts were disclosed)
   None

3C. Unpaid consultants for a company or supplier (The following conflicts were disclosed)
   None

4. Stock or stock options in a company or supplier (The following conflicts were disclosed)
   None
5. Research support from a company or supplier as a Principal Investigator (The following conflicts were disclosed)
   None
6. Other financial or material support from a company or supplier (The following conflicts were disclosed)
   None
7. Royalties, financial or material support from publishers (The following conflicts were disclosed)
   None
8. Medical/Orthopaedic publications editorial/governing board (The following conflicts were disclosed)
   None
9. Board member/committee appointments for a society (The following conflicts were disclosed)
   None

Each author must sign AND print or type his/her name, date and submit a separate form.

In addition, one BLINDED Conflict of Interest form (no author names used) should be submitted per manuscript with all author disclosures.

Angel Xiao		5/27/2025
Author Name (Print or Type)	Author Signature	Date
